# Sustained Delivery of Activated Rho GTPases and BDNF Promotes Axon Growth in CSPG-Rich Regions Following Spinal Cord Injury

**DOI:** 10.1371/journal.pone.0016135

**Published:** 2011-01-24

**Authors:** Anjana Jain, Robert J. McKeon, Susann M. Brady-Kalnay, Ravi V. Bellamkonda

**Affiliations:** 1 Neurological Biomaterials and Therapeutics, Wallace H. Coulter Department of Biomedical Engineering, Georgia Institute of Technology/Emory University, Atlanta, Georgia, United States of America; 2 Department of Cell Biology, School of Medicine, Emory University, Atlanta, Georgia, United States of America; 3 Department of Molecular Biology and Microbiology and Department of Neurosciences, School of Medicine, Case Western Reserve University, Cleveland, Ohio, United States of America; The University of Akron, United States of America

## Abstract

**Background:**

Spinal cord injury (SCI) often results in permanent functional loss. This physical trauma leads to secondary events, such as the deposition of inhibitory chondroitin sulfate proteoglycan (CSPG) within astroglial scar tissue at the lesion.

**Methodology/Principal Findings:**

We examined whether local delivery of constitutively active (CA) Rho GTPases, Cdc42 and Rac1 to the lesion site alleviated CSPG-mediated inhibition of regenerating axons. A dorsal over-hemisection lesion was created in the rat spinal cord and the resulting cavity was conformally filled with an *in situ* gelling hydrogel combined with lipid microtubes that slowly released constitutively active (CA) Cdc42, Rac1, or Brain-derived neurotrophic factor (BDNF). Treatment with BDNF, CA-Cdc42, or CA-Rac1 reduced the number of GFAP-positive astrocytes, as well as CSPG deposition, at the interface of the implanted hydrogel and host tissue. Neurofilament 160kDa positively stained axons traversed the glial scar extensively, entering the hydrogel-filled cavity in the treatments with BDNF and CA-Rho GTPases. The treated animals had a higher percentage of axons from the corticospinal tract that traversed the CSPG-rich regions located proximal to the lesion site.

**Conclusion:**

Local delivery of CA-Cdc42, CA-Rac1, and BDNF may have a significant therapeutic role in overcoming CSPG-mediated regenerative failure after SCI.

## Introduction

Physical injury to the spinal cord results in the damage and death of neurons and axons, leading to loss of both sensory and motor neuronal function. Injury triggers a cascade of events, including the migration of macrophages and activation of resident glial cells, microglia and astrocytes [1]. These cells express and deposit chondroitin sulfate proteoglycans (CSPGs) that contribute to regenerative failure by forming a repulsive barrier to growth cones [2], [3]. Besides CSPGs, myelin-associated inhibitory molecules including myelin-associated glycoprotein (MAG) [4], [5], Nogo [6], and oligodendrocyte myelin glycoprotein (OMgp) [7], located on the inner myelin sheath, are exposed after physical injury, further contributing to the inhibitory environment, thus impeding axonal growth. Therapeutic strategies aimed at overcoming these inhibitory regions usually target one of these proteins.

However, as more than one ‘inhibitory’ entity exists after spinal cord injury (SCI), we examined a strategy to alter the intrinsic axonal responses to inhibitory cues using an *in situ* gelling hydrogel system [8] to deliver constitutively active (CA) Rho GTPases and Brain-derived neurotrophic factor (BDNF). Our approach served to: (1) physically bridge the lesion, providing an axon growth permissive substrate for regenerating and sprouting nerve fibers, and (2) allow the local, slow release delivery of protein(s), such as CA-Rho GTPases or BDNF for a period of at least 2 weeks.

Rho GTPases, Cdc42 and Rac1, are responsible for the filopodial and lamellopodial extension of axonal growth cones [9], [10], whereas activation of Rho leads to growth cone collapse [10]. One of the main pathways through which Cdc42 promotes filopodial extension is the Neuronal Wiskott-Aldrich Syndrome Protein (N-WASP) and Actin-Related Protein 2/3 (ARP 2/3) Complex [11]. Activation of Cdc42 in turn leads to an immediate activation of Rac1 due to cross-talk [12]. The downstream effectors for Rac1, which activates actin polymerization are Insulin receptor substrate p53 and WASP famlily verprolin-homologous protein (WAVE) [13]. When either CA-Cdc42 and CA-Rac1, or inhibitors of Rho and its signaling pathways were transduced into neurons *in vitro*, neurites extend through inhibitory, CSPG-rich regions [14], [15], [16], [17]. Additionally, when inhibitors to Rho and ROCK were delivered *in vivo*, axonal regeneration was enhanced [18]. However, axon re-growth was not significant after C3 transferase, a bacterial toxin that inhibits Rho, was transduced [19]. To date, the roles of CA-Cdc42 and CA-Rac1 have not been examined *in vivo* after SCI. Therefore, to our knowledge this is the first study to provide controlled release of Cdc42 and Rac1 *in vivo*.

Distinct from altering growth cone sensitivity to inhibitory signals, it is important to promote neuronal survival and stimulate axon growth. Neurotrophins, such as BDNF, neurotrophin-3 (NT-3), and nerve growth factor (NGF), are neuroprotective *in vivo* [20], as well as stimulate axonal growth [21], [22]. We have previously demonstrated that local sustained delivery of BDNF is a potential strategy to promote axonal sprouting after SCI [8].

In this study, an agarose hydrogel scaffold embedded with lipid microtube delivery vehicles that provide local, sustained bioavailability *in vivo* was used to deliver CA-Cdc42, CA-Rac1, and BDNF after SCI. Our results demonstrated that there was *in vivo* release for at least 2 weeks, and all three treatments had an effect on the reduction of reactive astrocytes and CSPG deposition, and axonal growth within the inhibitory CSPG-rich regions. CA-Cdc42 significantly reduced the amount of reactive astrocytes and CSPG deposition, and treatment with CA-Rac1 and BDNF had a statistically higher number of axons growing within inhibitory CSPG-rich regions compared to the untreated control.

## Materials and Methods

### Ethics Statement

All animals were handled in strict accordance with good animal practices as defined by the relevant national and/or local guidelines. All animal procedures were approved (Protocol ID A04016) by the Georgia Institute of Technology's Institutional Animal Care and Use (IACUC) Committee.

### Fabrication and Loading of Lipid Microtubes for sustained release of CA-Rac1, CA-Cdc42 and BDNF

Lipid microtubes were fabricated as previously described [23], [24]. Briefly, 1,2-bis-(tricosa-10,12-diynoyl)-sn-glycero-3-phosphocholine (DC_8,9_PC, Avanti Polar Lipids, Alabaster, AL) was dissolved in 70% ethanol at a concentration of 1 mg/mL. The lipid was placed in a water bath (Thermo NESLAB, Portsmouth, NH) programmed to decrease from 50 to 20°C over 48 h and then stored at room temperature to facilitate self-assembly of lipid microtubes. Trehalose (18.9 mg/mL) was added to the microtubule solution and then dehydrated using a rotary evaporator. For the various treatments, 1 mg of dehydrated microtubes were loaded with BDNF (Millipore, Temecula, CA), CA-Cdc42, or CA-Rac1 and the loading concentrations were 500 µg/mL, 500 µg/mL and 230 µg/ml, respectively. The protein loaded microtubes were mixed into 2.6% agarose (w/v) at a 1∶1 volume ratio.

### Characterization of Microtubule Mediated Slow Release of BDNF *In Vitro*

Eighty microliters of 500 µg/mL BDNF was loaded into 2 mg of lipid microtubes by incubating the BDNF solution with dehydrated microtubes. The BDNF loaded microtubes were mixed into 2.6% agarose to obtain a microtubule concentration of 8.33 mg/mL in 1.3% concentration of agarose. Forty microliters of the BDNF loaded microtubes and agarose mixture was added into well of a 96 well plate, allowed to gel, and 150 µL of PBS was added to the above mixture. The 96 well plate was placed in a 37°C oven and after the first 24 hours, the 150 µL of phosphate buffered saline (PBS) was removed and replaced with 150 µL of fresh PBS. This method was followed every 48 hours for the following 13 days. The retrieved PBS solutions were stored in −20°C until they were ready to be analyzed for BDNF content. The BDNF released from the microtubes was quantified using the BDNF ELISA Sandwich Kit (Chemicon) and then the standard and sample wells were placed in the Synergy HT Micro Detection Microplate Reader (BIO-TEK Instruments, Inc., Winooski, VT) and the absorbance was read at 450 nm. A standard curve was obtained from the absorbances of the BDNF standards to determine the concentrations of the BDNF released in the PBS.

### Rhodamine Conjugation to BDNF for Characterization of Lipid Microtube Mediated Slow Release *In Vivo*

To permit visualization and characterization of BDNF's diffusion through spinal cord tissue, Rhodamine was conjugated to BDNF. The protocol provided with the EZ-Label™ Rhodamine Protein Labeling Kit (Pierce, Rockford, IL) that allows Rhodamine to be conjugated to most biomolecules using primary amines was followed to conjugate the Rhodamine to BDNF (BDNF/Rhodamine). The BDNF/Rhodamine was diluted to 500 µg/mL and then loaded to the microtubes as described above. The agarose/BDNF/Rhodamine loaded microtubes mixture was injected into the spinal cord as described below. After 2 weeks the spinal cords were retrieved and processed, as described in further detail below.

### TAT Rho GTPase Protein Expression and Purification

TAT Rho GTPase fusion proteins were prepared as previously described [25]. The pTAT-HA vector contains an N′ terminal 6-histidine leader followed by the 11 amino acid TAT protein transduction domain flanked by glycine residues, a hemaglutinin tag and a polylinker. Expression plasmids for constitutively active (CA) and dominant negative (DN) Rac1, RhoA and Cdc42 were kindly provided by Dr. Steven Dowdy (UCSD, CA) [26]. The expression plasmids were transformed into BL21 (DE3) pLysS competent cells (Novagen). A 100–200 ml LB overnight culture was started, then the entire volume was inoculated into 1 L of LB plus 200 mM IPTG and shaken for 5 hr at 37°C. The cell pellet was washed in PBS. Cells were resuspended in 1× PBS containing 2 µl/ml protease inhibitor cocktail (Sigma) and 1 µl/ml 1M benzamidine, sonicated on ice three times for 30 s and centrifuged at 4200 rpm for 25 min at 4°C. The supernatant was applied to 1 ml packed Talon Metal Affinity Resin (BD Biosciences, Clontech) to bind to the His-tag of the Rho GTPase fusion proteins and rocked overnight at 4°C. Beads were pelleted (1500 rpm, 3 min, 4°C) and washed three times with 10 ml 1× PBS/protease inhibitor cocktail/benzamidine followed by 3 additional washes with 10 ml 1× PBS. Protein was eluted from beads with 1× PBS containing 200 mM imidazole. Purified proteins were dialyzed into appropriate buffers for the experiment.

### Surgical Procedures

#### *In situ* gelling of Agarose-Protein Scaffolds in a Dorsal Over-hemisection Model *In Vivo*

Male Sprague-Dawley (Charles River Laboratory) rats weighing 190–230 grams were anesthetized with Nembutal®. The skin and muscle were opened to expose the thoracic vertebrae T8–T10 and the underlying spinal cord was exposed. A modified dorsal-over hemisection was made by creating a 2 mm×2 mm×1.5 mm deep cavity thereby removing the dorsal portion of the corticospinal tract (CST). Four microliters of a protein loaded microtubes/agarose solution was injected into the cavity. A cooling system was used to gel the agarose scaffold containing the microtubes loaded with protein [8]. The muscle and the skin were closed in layers. The animals were given Buprenoprhine post-surgery. The animal bladders were manually expressed twice a day until urinary function was recovered. There were 6 animals in each group.

#### Anterograde Neuronal Tracer Injection into the Corticospinal Tract

Four weeks post-injury, biotinylated dextran amine (BDA, Invitrogen) was bilaterally injected into the motor cortex to trace the axons in the corticospinal tract (CST). The animals were anesthetized with 2% isoflurane and maintained through the surgery at 0.5% isoflurane. The skin and the periosteum were opened to expose the skull. Three injection sites, located within each hemisphere 2 mm from the midline and 1 mm apart from each site, were used to inject 0.5 µL of BDA at each site over 2 min. Six weeks post-injury (two weeks after BDA injection), the animals were anesthetized with ketamine (1 mL/kg), xylazine (0.17 mL/kg), and Acepromazine (0.37 mL/kg), transcardially perfused with 4% paraformaldehyde, the spinal cords were retrieved, and incubated in 4% paraformaldehyde overnight and then stored in PBS containing 0.01% sodium azide.

#### Immunohistochemistry and Histological Evaluation of Explanted Spinal Cords and Hydrogels

Serial sagittal sections (25 µm) of the spinal cords were cut on a cryostat (Leica CM 300, Leica, Bannockburn, IL) and mounted onto glass slides. To visualize reactive astrocytes and microglia/macrophages, and labeled axons, a triple-stain was performed using anti-rabbit glial fibrillary acidic protein (GFAP) (Chemicon), ED-1 (mouse anti-rat CD68, Serotec, Raleigh, NC), and BDA. The sections were incubated in 4% goat serum in 0.5% Triton-X 100 for 1 hour. GFAP (1∶1000) and ED-1 (1∶1000) in 4% goat serum and 0.5% Triton-X 100 were added to the sections and incubated at 4°C overnight. The secondary antibodies, Alexa Fluor 350 goat anti-rabbit IgG (1∶200 dilution) (Molecular Probes, Eugene, OR), Alexa Fluor 594 goat anti-mouse (1∶200 dilution) (Molecular Probes), and Alexa Fluor 488 Streptavidin (1∶200) were added for GFAP, ED-1, and BDA, respectively, and allowed to incubate for 1 hour at room temperature. The sections were rinsed twice with 0.1M PBS and covered with glass coverslips.

A triple stain was also done with BDA, GFAP and CS-56, an antibody to identify growth inhibitory CSPGs (Sigma, St. Louis, MO). Spinal cord sections were incubated overnight at 4°C with anti-GFAP and CS-56 (1∶250) in 4% goat serum and 0.5% Triton-X 100. The secondary antibodies, Alexa Fluor 350 goat anti-rabbit (1∶200), Alexa Fluor 594 goat anti-mouse IgM (1∶200), and Alexa Fluor 488 Streptavidin (1∶200) were added as described above. In addition, to help visualize the axons and neurons at the injury site a double stain with BDA and anti-mouse Neurofilament 160 kDa (NF-160 kDa, Sigma) was performed as described above. An anti-rabbit calcitonin gene related peptide (CGRP, Chemicon) was used to identify sensory fibers around the lesion site. All images were taken on the Zeiss Axioskop 2 Plus microscope (Zeiss, Thornwood, NY).

### Quantitative Analysis of Cellular, Molecular, and Axonal Response to Scaffold and Protein

#### Methodology for GFAP and CS-56 Analysis: Identifying the inhibitory regions

The methodology to quantify the fluorescent intensity of GFAP and CS-56 images has been previously published [8]. All images were taken at the same exposure time and conditions. A minimum of 60 images (4 images/spinal cord section×5 spinal cord section/each animal×3 animals) per experimental group was utilized to obtain the overall average intensity profiles for each staining representing 1800 different individual intensity profiles per condition. The overall average intensity profiles for both GFAP and CS-56 as function of distance from the interface was compared between experimental groups.

#### Quantification of Average Lesion Area

The lesion area was identified as the cavity surrounded by the GFAP^+^ stained boundary. Ten sections that were located at the epicenter of the lesion were used to determine the lesion area. The lesion area was measured using ImagePro software and the average was then calculated for each of the conditions.

#### Quantification of ED-1^+^ Cells

The number of ED-1^+^ cells in and around the lesion site was quantified. Two 10× images were taken of each section using an Olympus digital camera attached to the Zeiss Axioskop 2 Plus microscope. ImagePro software (Media Cybernetics, Carlsbad, CA) was used to count the number of cells that were present. Two parameters, (1) the fluorescent intensity and (2) cell diameter, were kept constant for all the images of the different conditions when counting the cells.

#### Quantification of BDA^+^ Axons

Spinal cord sections stained for BDA^+^ axons were imaged at 10× on the Nikon Eclipse 80i upright microscope using a Microfire CCD camera (Optronics, Goleta, CA) that interfaced with the Neurolucida software (MicroBrightField Bioscience, Williston, VT) to obtain a montage of each section. The images were used to measure axon distance from the lesion site for all of the conditions. The distance between the 3 or more closest axons, with dystrophic endbulbs, and the lesion site was measured using ImagePro Software.

The beginning of the proximal side of the lesion was marked as 0 mm and every millimeter was marked and ‘binned’ up to a distance of 4 mm rostral to the lesion site. The number of axons 4 mm proximal to the lesion site was considered to be the original number of axons in the CST and is written as total number of axons at 4mm in the equation below. The number of BDA^+^ axons, from 0 to 1 mm, 1 to 2 mm, 2 to 3 mm, and 3 to 4 mm, proximal to the injury site was counted using 10× montage images. The percent of axonal outgrowth was determined by the following equation:

(Number of axons at specific distance range/Total number of axons at 4 mm) * 100

#### Distance of Axonal Growth Through and Within Inhibitory Regions

CSPGs are present in the glial scar around the lesion area at high levels in the immediate vicinity of the lesion, but extending up to 400–500 microns away from the lesion border. The fluorescent intensity values of CS-56 stained regions were used to identify these ‘inhibitory regions’. To determine the degree to which regenerating axons where able to overcome CSPG-mediated inhibition, BDA^+^ axons were placed in three categories: (1) axons that stopped before the CS-56^+^ inhibitory region, (2) axons crossed the proximal interface of the inhibitory region but stopped within the inhibitory region, and (3) axons that crossed the distal interface of the inhibitory region and grew past it; and counted. A percent of axons in each region was determined by the following equation:

(No. of axons in region/Total number of axons in all 3 regions) *100

The axons that had stopped within the inhibitory region mentioned in the section above were then measured to determine the distance extended after crossing the proximal interface. The shortest total distance extended by the axons within the inhibitory region was measured and then averaged. The length of all the axons that stopped their outgrowth within this region was measured and then averaged for each condition. The percent of the distance extended by the axons within the CS-56 region was calculated by the following equation:

(Average Axonal Distance Extended/Total Length of Inhibitory Region) *100

### Statistical Analysis

GFAP, CS-56, and NF-160: The area under the curve of the overall average intensity profiles was determined for four different distance ‘bins’: 0 to 100 µm, 100 to 200 µm, 200 to 300 µm, and 300 to 400 µm from the interface extending into the spinal cord. The area under the curve of the profiles was statistically compared by ANOVA and the post-hoc test Tukey's test (P<0.05). To determine if there was significant difference amongst the conditions and between different groups, ANOVA and Tukey's test (P<0.05) was performed for all other analysis.

## Results

### Release Characteristics of BDNF *In Vitro* and BDNF Conjugated to Rhodamine *In Vivo*

**Figure 1:**
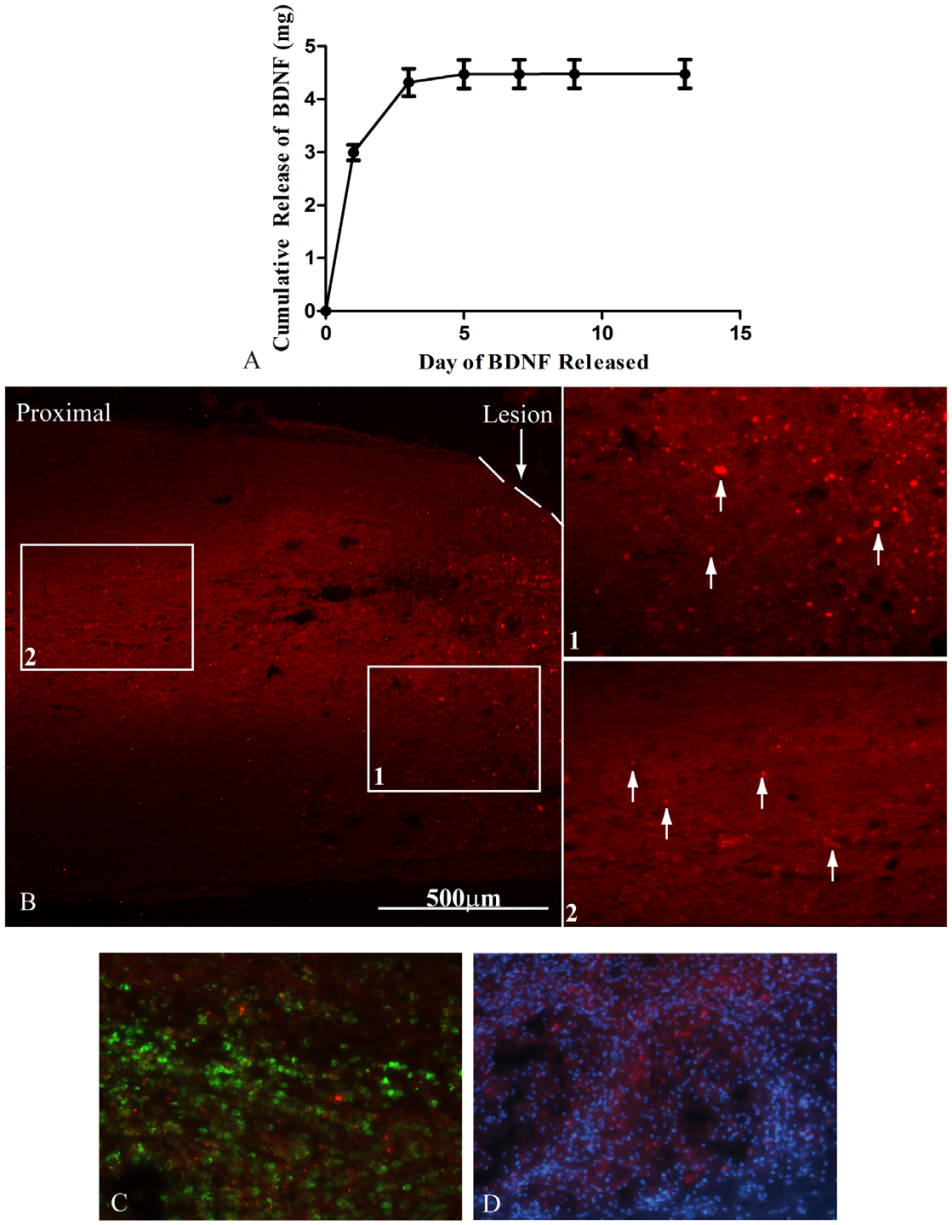
*In Vitro and In Vivo* Diffusion of BDNF. **A**. *In vitro* release assay of BDNF over the first 2 weeks. The graph shows that an initial burst released 4 µg of BDNF within the first 3 days. An average of 3 ng/day of BDNF was released for the following 11 days. The data represent mean ± SEM **B**. An image of the proximal region of a spinal cord section after delivery of BDNF/Rhodamine at 4×. The 20× images labeled 1 and 2 are outlined with white boxes in A and demonstrate that BDNF/Rhodamine diffused approximately 2 mm proximal to the lesion site. The dashed line represents the interface between the spinal cord and scaffold. White arrows indicate BDNF/Rhodamine. **C**. A 20× image of ED-1^+^ cells (green) and BDNF-Rhodamine (red). The image shows some overlap demonstrating engulfment of the BDNF-Rhodamine by the ED-1^+^ cells. **D**. A 20× image of DAPI (blue) and BDNF-Rhodamine (red) demonstrates that not all of the BDNF-Rhodamine is taken up by resident and migrating cells.

An *in vitro* release assay for BDNF loaded microtubes was performed. Approximately 3 µg of BDNF was released within the first 24 h. A cumulative of 4 µg of BDNF was released within the first 3 days. Figure 1A shows that approximately 3 ng/day of BDNF was released for the subsequent 11 days. Microtubes loaded with BDNF/Rhodamine embedded in the agarose were injected into the spinal cord cavity.

Lipid microtubes loaded with BDNF/Rhodamine embedded in the agarose were injected into the spinal cord cavity. For this experiment only, the spinal cords were retrieved two weeks post-injury and sectioned. BDNF/Rhodamine can be seen in the spinal cord tissue between 1 to 2 mm proximal to the lesion site (Fig. 1B). Insets 1 and 2 show magnified images of BDNF/Rhodamine. White arrows indicate some of the BDNF/Rhodamine 1 mm and 2mm from the lesion site. Figure 1C demonstrates that some of the ED-1^+^ cells (green) overlap with the BDNF-Rhodamine. The image shows some co-localization demonstrating engulfment of the BDNF-Rhodamine by the ED-1^+^ cells. The BDNF-Rhodamine was double labeled with DAPI to visualize cell nuclei (blue) in Figure 1D and demonstrate that the neurotrophin is present extracellularly and has the potential to be taken up by both resident and migrating cells.

### Cellular and Molecular Response Around Lesion Site

**Figure 2:**
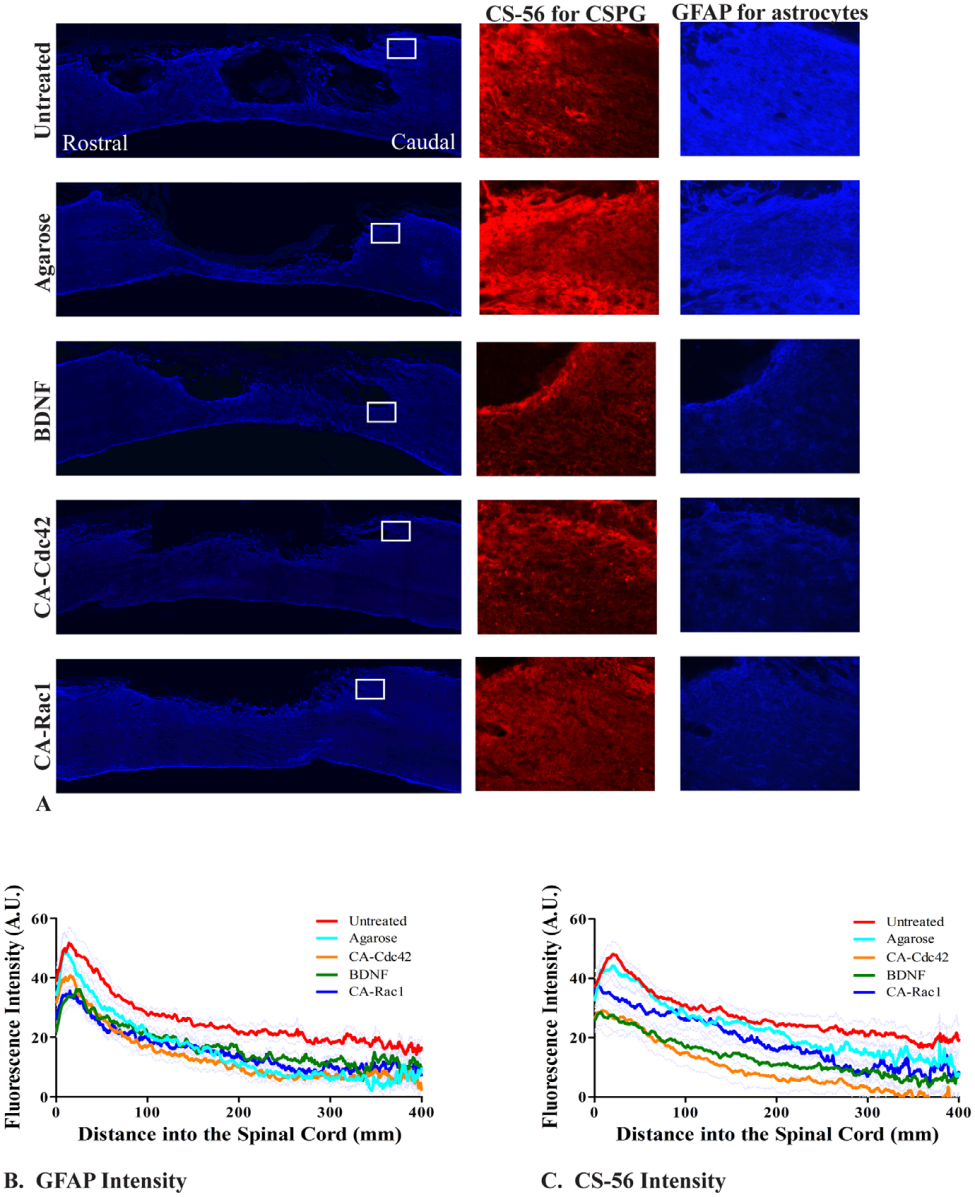
GFAP and CS-56 expression at the lesion site. **A**. Images of the lesion site for the controls and treated conditions that show the GFAP stain for reactive astrocytes at 10×. The white box represents the focused image (20×) to the right of CS-56 (red) and GFAP (blue), respectively. The rows of images are untreated spinal cord, Agarose, BDNF, CA-Cdc42, and CA-Rac1. **B**. The GFAP intensity for BDNF, CA-Cdc42, and CA-Rac1 compared to the untreated control is significantly less. CA-Cdc42 was significantly less intense compared to the agarose control. The data represent the mean. One way ANOVA and Tukey's test were used to statistically analyze the data. The gray lines represent the ± SEM. (p<0.05 BDNF, CA-Cdc42, and CA-Rac1 compared to untreated control, p<0.05 CA-Cdc42 compared to agarose control). **C**. The CS-56 intensity was significantly lower in spinal cords treated with CA-Cdc42 and BDNF compared to the untreated and agarose controls. The data represent the mean. The gray lines represent ± SEM. (p<0.05 CA-Cdc42 and BDNF compared to untreated and agarose controls).

Six weeks post injury, regions with reactive astrocytes (high GFAP intensity) are generally correlated with increased CSPG deposition (CS-56 intensity) (Fig. 2A). Untreated controls have high levels of GFAP and CS-56. Use of the agarose hydrogel alone caused a reduction in reactive astrocytes (GFAP). When quantified (Figs. 2B–C), the GFAP and CS-56 intensity is statistically lower in CA-Cdc42, CA-Rac1, and BDNF treated animals as compared to the untreated controls. In addition, CA-Cdc42 delivery resulted in significantly lower GFAP and CS-56 intensity compared to the agarose control.

**Figure 3:**
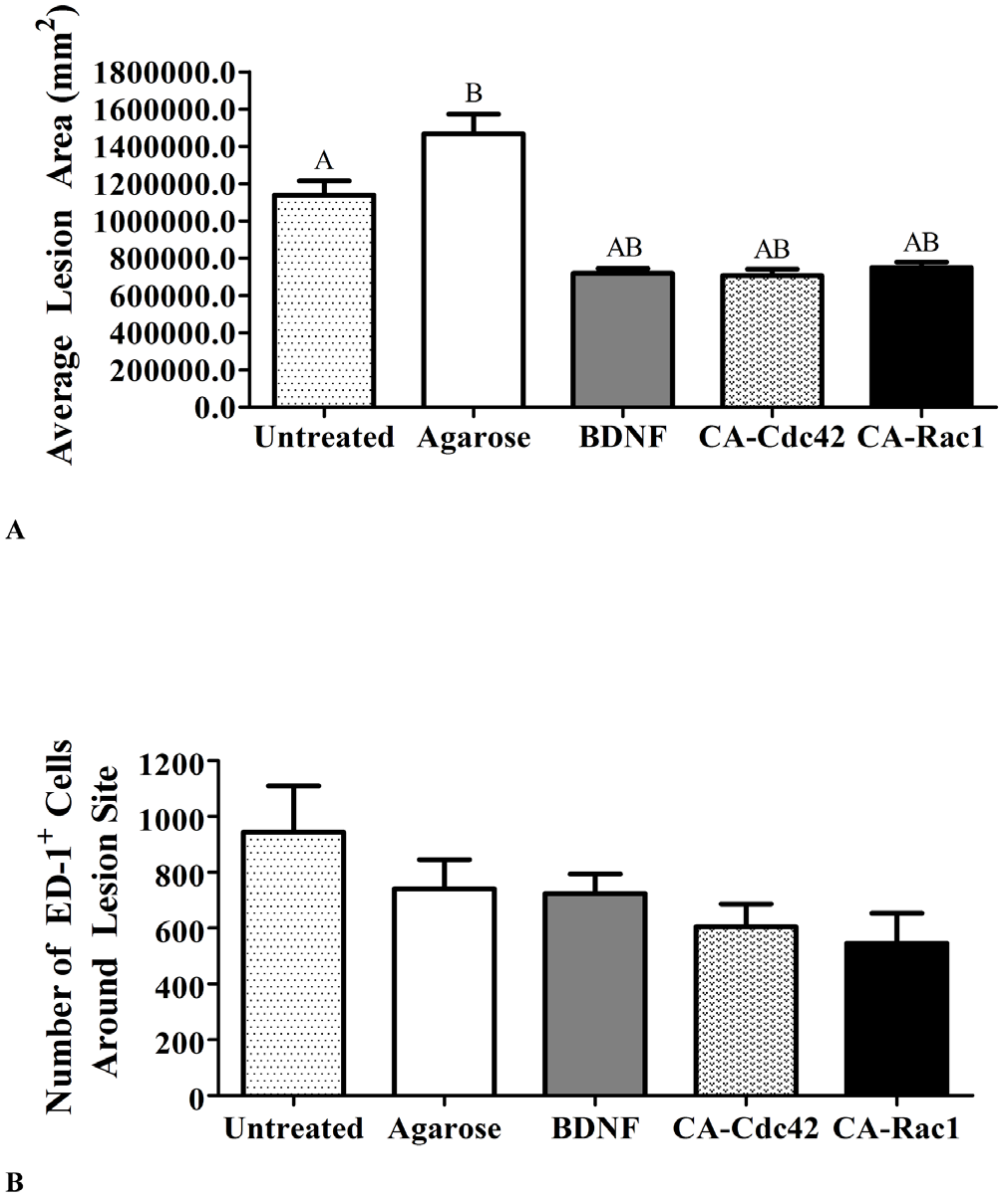
Quantification of lesion area and ED-1^+^ cells. **A**. Average lesion area of the controls and treated spinal cords. The average lesion area was statistically smaller in all of the treated conditions compared to the untreated and agarose controls. The data represent the mean ± SEM. One way ANOVA and Tukey's test were used to statistically analyze the data. (A p<0.05 compared to untreated control and B p<0.05 compared to agarose control). **B**. Comparison of the number of ED-1^+^ macrophages/reactive microglia at the interface. This bar graph shows that there was not a statistical difference in the number of macrophages/reactive microglia in the treated animals compared to the untreated and agarose controls. The data represents mean ± SEM.

The average lesion area was measured for all the experimental groups to determine if the treatments caused a greater secondary injury. The average lesion area for the untreated and agarose controls was greater than 1 mm^2^, which was significantly larger than all of the treated groups, BDNF, CA-Cdc42, and CA-Rac1 that had an area approximately 0.7 mm^2^ (Fig. 3A). These data indicate that treatment results in a smaller lesion cavity than even in the agarose control.

Besides staining for GFAP and CS-56 for a cellular and molecular response at 6 weeks, reactive microglia/macrophages in and around the lesion site were quantified by counting the number of ED-1^+^ cells (Fig. 3B). A statistical difference between experimental and control treated animals was not observed.

### Sprouting Axonal Tracts

**Figure 4:**
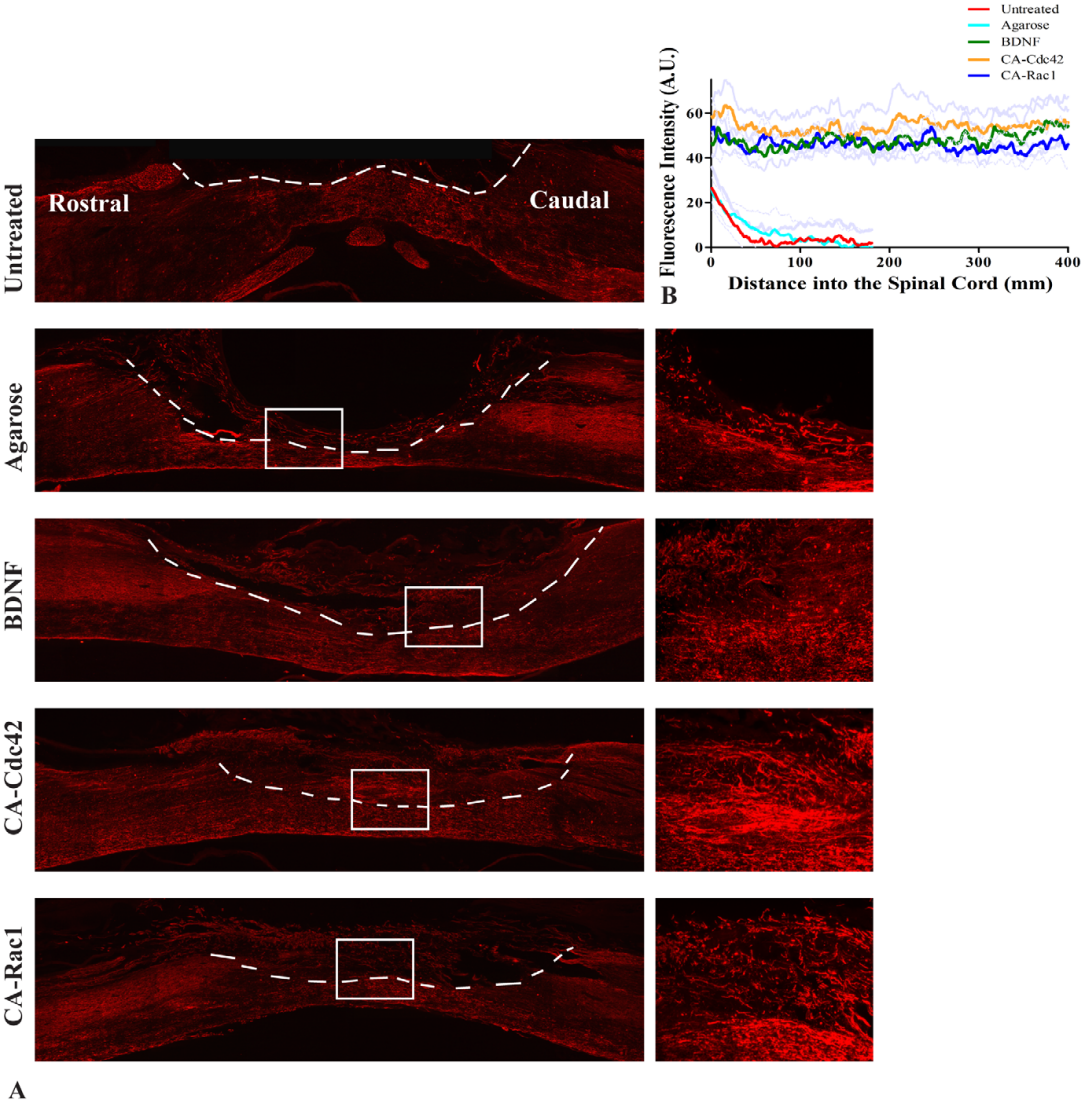
NF-160^+^ fluorescent images. **A**. Representative images for the controls and treated conditions with NF-160 stain at 10×. The interface between the spinal cord tissue and scaffold is identified with a white dashed line. To the right of each image, is a 20× image of the area outlined with a white box. The rows show Untreated, Agarose, BDNF, CA-Cdc42, CA-Rac1, respectively. In the treated conditions, NF-160^+^ axons are in the scaffold-filled cavity, where as NF-160^+^ axons were not observed in the controls. **B**. Quantitative analysis of NF-160 intensity for the stained spinal cords. The spinal cords treated with CA-Cdc42, CA-Rac1, and BDNF had significantly higher fluorescent intensity and also had extended further into the scaffold-filled spinal cord cavity than the untreated and agarose controls.

**Figure 5:**
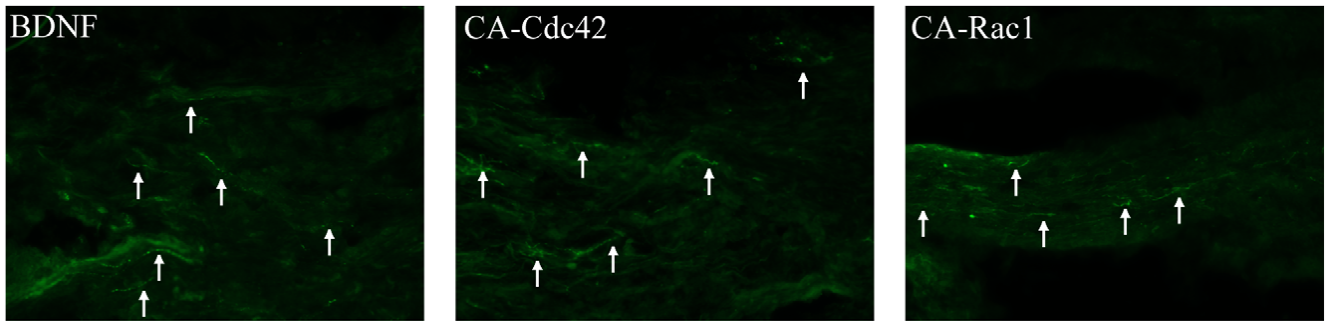
Fluorescent images of CGRP^+^ axons extending through the agarose hydrogel in the treatment conditions, BDNF, CA-Cdc42, and CA-Rac1. Arrows point at some of the CGRP^+^ axons in the 20× images.

NF-160 was used to observe whether there were axons located in the hydrogel scaffold and the glial scar (Fig. 4A). The NF-160 stain was quantified using the custom MATLAB program utilized for the GFAP and CS-56 analysis. In the untreated and agarose controls, the NF-160 staining did not have measurable intensity beyond 150 µm into the scaffold (Fig. 4B). However, the treatment groups, BDNF, CA-Cdc42, and CA-Rac1, had a significantly higher fluorescent intensity compared to the controls and the intensity levels remained consistent throughout the scaffold. NF-160^+^ neurons had extended significantly further through the hydrogel scaffold in the treated animals compared to the untreated and agarose controls (Fig. 4B). A significant difference was not observed amongst the treatment groups. NF-160 staining identifies a variety of axons within the spinal cord. An antibody against the sensory neuropeptide CGRP was used. As it was observed that the scaffolds in the treatment groups (BDNF, CA-Cdc42, and CA-Rac1) were positive for NF-160, an antibody against CGRP was used to identify the type of axons. It was observed that CGRP^+^ sensory axons were present in the treated hydrogel scaffolds, which can be seen in the fluorescent images (Fig. 5). Therefore, some of the NF-160^+^ staining are CGRP^+^ sensory axons within the hydrogel scaffold.

### BDA^+^ Axons are Present in Glial Scar after Treatment

**Figure 6:**
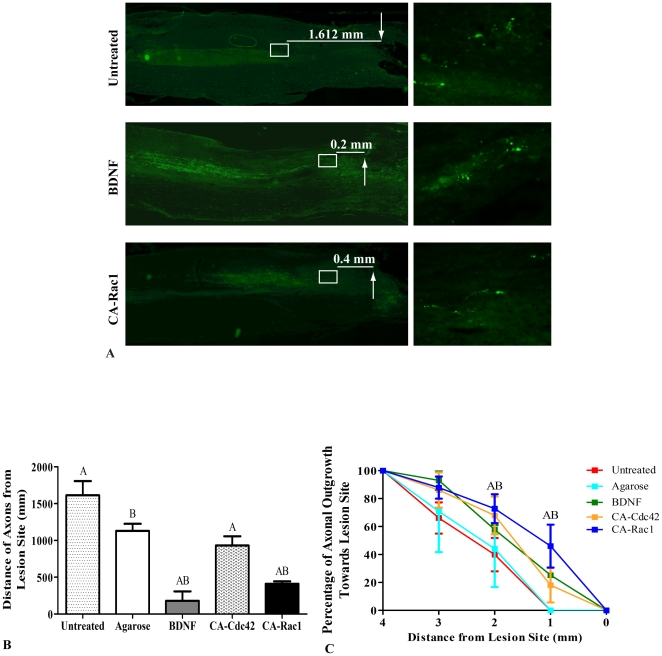
BDA^+^ axons in CST. **A**. Fluorescent images of the untreated control, BDNF and CA-Rac1 treatment conditions at 10×. White box represents the area of the image shown to the right at 40×. The arrows represent the beginning of the lesion site. The average distance of the closest axons to lesion site is written. The images on the right show the closest axons to the lesion site. **B**. Axonal retraction from the lesion site in the treated and control spinal cords. The distance of 3 or more axons from the lesion site in each image was averaged. All the treated cords had axons within 1 mm of the lesion site and were significantly closer to the lesion site compared to the untreated and agarose treated cords. BDNF and CA-Rac1 were the most effective treatments and were within 400 µm to the lesion site. The data represents mean ± SEM. One way ANOVA and Tukey's test were used to statistically analyze the data. (A p<0.05 compared to untreated controls and B p<0.05 compared to agarose control). **C**. Percent of axonal outgrowth towards the lesion site. The percent of axons was measured from 4 mm proximal to the beginning of the lesion site. The graph shows that 2 mm from the lesion site, the 68–72% of the axons are present in the conditions treated with CA-Cdc42 and CA-Rac1 compared to the untreated and agarose controls, which had significantly lower percent of axons (45–50%). One millimeter away from the lesion site, there were not any axons in the control conditions, where as there were 35–45% of the axons in the spinal cords treated with CA-Rac1. The data represents mean ± SEM (A p<0.05 compared to untreated control and B p<0.05 compared to agarose).

Axons in the CST, originating in the motor cortex, located in the dorsal column were severed during the dorsal-over hemisection. The axons were labeled with BDA to visualize their location in regards to the lesion site (Fig. 6A). The images on the right show the closest axons to the lesion site. Axons with dystrophic endbulbs retracted over 1 mm from the beginning of the lesion site in both the untreated and agarose conditions (Fig. 6B). All treatments showed a reduction in axonal retraction compared to controls. However, in animals with BDNF and CA-Rac1 treatment, axons were within 400 µm proximal to the lesion site, which is significantly closer compared to the other conditions. These data suggest that axonal dieback or retraction was altered by BDNF and CA-Cdc42 and CA-Rac1 treatment of the lesion cavity.

In addition to measuring the distance from the lesion site to the closest axons, the percent of axons every millimeter from 4 mm proximal to the lesion site was determined (Fig. 6C). The number of axons that were present at 4 mm was considered to be the total number of axons present both before and after the lesion. By 2 mm, there were greater percentage of axons in the treated groups, BDNF, CA-Cdc42, and CA-Rac1, which were 58%, 68%, and 73%, respectively, compared to the 40 and 44% of untreated and agarose controls (Fig. 6C). Between the ranges of 0 to 1 mm, there were 20–35% axons near the lesion in the treatment groups, where as in the controls, axons were not present within 1 mm proximal to the lesion site.

### Crossing of Axons through Inhibitory Regions *In Vivo*

**Figure 7:**
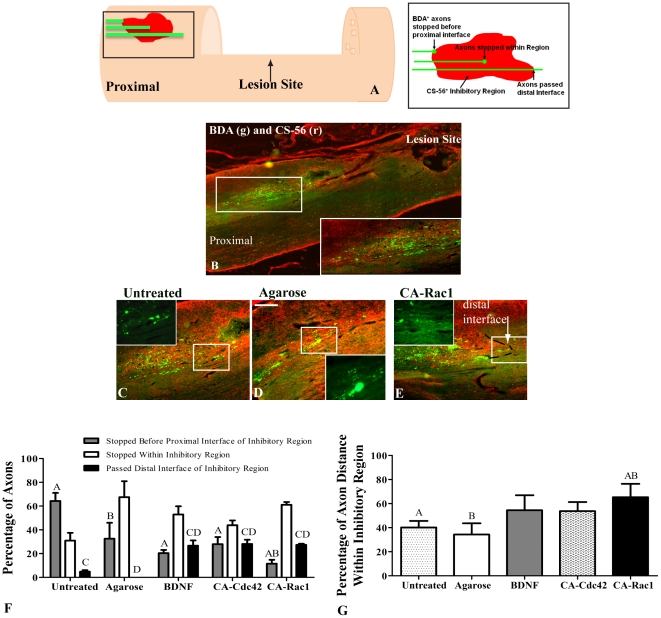
Characterization of CST^+^ axons extending through CS-56^+^ regions proximal to lesion site. **A**. Schematic of the inhibitory regions quantified is depicted with the inhibitory region magnified in a schematic to the right. For axon quantification in CS-56^+^ inhibitory regions, axons were placed into 3 categories: axons that stopped before the proximal interface of the inhibitory region, axons that stopped within the inhibitory region, and axons that passed the distal interface of the inhibitory region. **B**. 10× image of BDA (green) and CS-56 (red) at the proximal side of the lesion site. The white box is magnified at the bottom right corner demonstrating how the BDA^+^ axons stall at the CS-56^+^ regions. **C–E**. 10× images of BDA^+^ axons and CS-56^+^ inhibitory regions proximal to the lesion site. The white box represents the image overlaid (40×) with BDA. **C and D** show the axons stopped in the middle of the CS-56^+^ regions for the controls, where as in **E**, it can be seen that with CA-Rac1 treatment the axons cross the distal interface of the inhibitory region shown with a black dotted line as indicated with a white arrow. **F**. Percentage of axons in CS-56^+^ inhibitory regions. A significantly higher percentage of axons crossed the distal interface of the inhibitory region in the BDNF, CA-Cdc42, and CA-Rac1 compared to the untreated and agarose controls. Also, the axons in the controls stopped at the proximal interface of the inhibitory region at a significantly higher percentage than in the spinal cords treated with CA-Rac1. **G**. In the spinal cords treated with CA-Rac1, the axons extended a significantly further distance within the inhibitory region than the untreated and agarose controls. One way ANOVA and Tukey's test were used to statistically analyze the data. (A and C p<0.05 compared to untreated control and B and D p<0.05 compared to agarose control). The data represents mean ± SEM.

CSPG containing regions (CS-56 positive) were identified proximal to the lesion site in the treated and control groups. The area was considered to be inhibitory if the CS-56 pixel intensity values were within the range of those in the glial scar bordering the lesion (Fig. 7). The numbers of axons which traversed the region, stopped within the inhibitory region, or stopped proximal to the inhibitory region interface were counted. The percentage of axons that stopped in each of the 3 regions was determined (Fig. 7E). All three experimental groups had significantly higher percentage of axons that traversed the distal interface of the inhibitory regions compared to the control groups. The animals treated with CA-Rac1 had the highest number of axons that crossed the proximal interface of the inhibitory region compared to the untreated or agarose-treated animals. This can be seen in Figure 7B–D. However, in all the conditions, a high percentage of axons, 45–65%, stopped within the inhibitory region (Figure 7F).

## Discussion

This study was performed to determine whether utilizing the hydrogel/microtubule scaffold delivery system to deliver BDNF, CA-Cdc42, or CA-Rac1 would decrease the sensitivity of growth cones to CSPGs, and promote axonal growth through CSPG-rich regions at the lesion site. First, the use of the *in situ* gelling hydrogel allows the lesion cavity to be conformally filled. Second, the lipid microtubes allow slow release of therapeutics from the hydrogel over time. Third, this strategy allows neuroprotective or axonal migration stimulators to be delivered locally over time.

Lipid microtubes were embedded within *in situ* gelling hydrogels that released CA-Cdc42, CA-Rac1, or BDNF over time. The sustained release profile of BDNF was characterized *in vivo*. Two weeks post-implantation, BDNF/Rhodamine is present around the lesion site, demonstrating at least 2 week release, as well as diffusion 2 mm from the lesion site (Fig. 1). This represents a critical period due to the formation of the glial scar and axonal retraction from the lesion site. Therefore, our delivery system affects the inhibitory response and axonal regrowth due to the proteins being available in the microenvironment and potentially taken up by microglia, macrophages, astrocytes, as well as neurons. The Rho GTPases are likely to display similar release characteristics from the microtubes due to the molecular weight of BDNF and the Rho GTPases both being approximately 25 kDa. Previous data from our laboratory demonstrated that the molecular weight determines the release characteristics from the microtubes and the neurotrophins are bioactive after release for at least two weeks *in vitro* [23], [24].

Animals treated with CA-Cdc42, CA-Rac1, and BDNF had significantly lower expression of GFAP and CSPGs than untreated controls. BDNF and Cdc42 treated spinal cords had the greatest reduction in CS-56 fluorescent intensity suggesting a reduction in CSPGs. It has been shown that Rho-associated kinase (ROK), a downstream effector of Rho, phosphorylates GFAP [27]. Cross-talk among the Rho GTPases has been well established, where activation of Cdc42 and Rac1 causes down-regulation of Rho. Therefore, if Rho is down-regulated due to the delivery of CA-Cdc42, CA-Rac1, and BDNF, then ROK may also be down-regulated, which could influence the phosphorylation of GFAP. Some studies have used chABC to digest the CSPGs in the inhibitory environment [28], [29], [30], [31]. This is a so called “extrinsic” strategy. While promising, there may be other inhibitory entities in astroglial scar tissue, such as the myelin inhibitory proteins. Therefore, the advantage of the ‘intrinsic’ strategy aimed at altering intracellular signaling that regulates growth cone sensitivity to inhibitory cues is that it may act independently of which inhibitors are present in the lesion. It was seen in Figure 2 and Figure 3, that the agarose control group had GFAP and CS-56 intensities and lesion volume that were statistically higher than the treated conditions. However, agarose statistically does not elicit a higher astrocytic response or CSPG deposition compared to the injured control. Also, Figure 3B demonstrates that the number of microglia and macrophages, counted using ED-1^+^ stain, is not higher compared to the other conditions. We believe that agarose is a neutral scaffold. That it does not prohibit nor promote axonal growth or increase the immune/inflammatory response.

In addition to the initial injury, secondary injury leads to the increase in the lesion size. It is important that the therapeutic strategies do not enhance lesion size. In a study using anti-transforming growth factor-β, although the antibody reduced astorycytosis, the lesion size increased [32]. In this study, the lesion area was measured to observe whether there was a similar occurrence. A difference in the reactivity of the astrocytes was observed in the treatment conditions, therefore, we wanted to quantify the lesion. However, the treated groups, BDNF, CA-Cdc42, and Rac1, decreased the lesion area compared to the controls. This suggests that BDNF, CA-Cdc42, and Rac1 may have influenced the reactivity and migratory response of the microglia and macrophages, which led to a decrease in inflammatory cytokine production.

Numerous NF-160^+^ axons were present in the treated animals. CGRP^+^ sensory axons infiltrated the inhibitory glial scar and the scaffold compared to the untreated and agarose controls. *In vivo* studies have shown that BDNF helps stimulate axonal outgrowth of sensory fibers [21], [22]. In our study, BDNF and the Rho GTPases, CA-Cdc42 and CA-Rac1, aid in axonal outgrowth of the sensory fibers. After injury, axonal retraction occurs for both ascending and descending tracts. Our data suggest that the retracted axons in the ascending sensory tracts extended through the glial scar surrounding the lesion site distal to the hydrogel/microtube filled spinal cord cavity due to the delivery of Cdc42, Rac1, and BDNF. This demonstrates that delivery of Cdc42, Rac1, and BDNF may have a therapeutic effect on axonal outgrowth after injury. The dosage of the three proteins may not have been at an optimal concentration or duration to stimulate axonal growth in the CST, however, the amount of protein was sufficient to promote growth of ascending sensory tracts.

The effect of BDNF, CA-Cdc42, and CA-Rac1 on axonal infiltration into the glial scar and scaffold-filled lesion was analyzed using the anterograde tracer BDA. Although BDA^+^ axons were not located within the scaffold-filled lesion, in the treatment conditions axons were present within the glial scar, in the inhibitory CSPG-rich regions, which was significantly increased from the untreated and agarose controls. The Rho GTPases, as well as BDNF, influence actin cytoskeleton dynamics by aiding in actin polymerization. These treatments may have reduced retraction or altered dieback of axons in the treated conditions to allow axons to extend toward the lesion site despite the presence of the CSPG inhibitory regions. Rather than promote actin polymerization, CA-Cdc42, CA-Rac1, and BDNF may have hindered actin depolymerization reducing the amount of axonal retraction. Therefore, the axons in the treated groups remained closer to the lesion site compared to the controls.

CSPG inhibitory regions, identified by the fluorescent intensity of CS-56 were observed proximal to the glial scar in the spinal cord at similar intensity values. When the astrocytes become reactive after injury, these cells deposit CSPGs creating a glial scar. The presence of the CSPG-rich inhibitory regions and the effects of the Rho GTPases and BDNF on axonal growth proximal to the lesion site, suggests that it is imperative to deliver therapeutics that block inhibitory signals not only into the cavity, but also to areas proximal to the lesion site, thereby increasing the number of axons extending towards and possibly through the lesion site.

Quantification of GFAP, CS-56, BDA, and NF-160 stains demonstrated a significant difference between the treatments and the control groups. Treatment with CA-Cdc42, CA-Rac1, and BDNF demonstrated NF-160^+^ axons through the hydrogel scaffold, a reduction in GFAP and CS-56 fluorescent intensity, thereby reducing astrocytes reactivity and CSPG deposition, and a higher percentage of BDA^+^ axonal extension towards the lesion site. This suggests that the Rho GTPases and BDNF may influence the inflammatory response possibly affecting the macrophage/microglia and astrocyte responses and neuronal axonal extension. The double combination of CA-Cdc42/CA-Rac1 or delivery of CA-Cdc2, CA-Rac1, and BDNF in combination was not performed in this study due to cytotoxic effects that were observed in the neurons when CA-Cdc42 and CA-Rac1 were transduced together [17]. The NF-160^+^ and CGRP^+^ axonal growth also demonstrates that local delivery of Rho GTPases and BDNF promotes axonal infiltration through CSPG-rich regions and into the lesion site filled with the hydrogel substrate after SCI. These findings suggest that there exists a significant potential for strategies aimed at modulating Rho GTPases alone, and in combination with each other or BDNF to overcome CSPG-mediated inhibition after SCI.
